# Conjugated Linoleic Acid: good or bad nutrient

**DOI:** 10.1186/1758-5996-2-62

**Published:** 2010-10-30

**Authors:** Daniela C Gonçalves, Fabio S Lira, Luiz C Carnevali, Jose C Rosa, Gustavo D Pimentel, Marília Seelaender

**Affiliations:** 1 Institute of Biomedical Sciences, University of São Paulo, São Paulo, Brazil; 2 Department of Physiology, Universidade Federal de São Paulo (UNIFESP), São Paulo/SP, Brazil

## Abstract

Conjugated linoleic acid (CLA) is a class of 28 positional and geometric isomers of linoleic acid octadecadienoic.Currently, it has been described many benefits related to the supplementation of CLA in animals and humans, as in the treatment of cancer, oxidative stress, in atherosclerosis, in bone formation and composition in obesity, in diabetes and the immune system. However, our results show that, CLA appears to be not a good supplement in patients with cachexia.

## 

Conjugated linoleic acid (CLA) is a class of 28 positional and geometric isomers of linoleic acid octadecadienoic [1]. CLA is produced by ruminants, from linolenic (18:3) and linoleic (18:2) unsaturated fatty acids obtained in the diet. Therefore, the natural sources of these substances are dairy products and red meat. Despite of the variety of geometric isomers of linoleic acid, they are present in nature mainly in (18:2) cis-9, trans-11 and (18:2) trans-10, cis-12 forms [2].

Currently, it has been described many benefits related to the supplementation of CLA in animals and humans, as in the treatment of cancer, oxidative stress, in atherosclerosis, in bone formation and composition in obesity, in diabetes and the immune system [3]. However, the most studied effect in relation to CLA supplementation is changes in lipid metabolism not only in animals but also in humans [4].

Although several studies show a beneficial effect of CLA supplementation on immune system parameters, data showing a relationship between CLA and a possible improvement of the are still scarce. The few found work related to CLA supplementation and decrease in muscle proteolysis is directly related to the development of cachexia. Yang and Cook [5] showed that CLA supplementation reduced weight loss, and a reduction of plasma TNF-α in cachetic rats induced by injection of LPS (Lipopolysaccharide - inducing the production of TNF-α by macrophages).

Graves et al. [6] showed that supplementation with CLA in cachetic rats decreased the muscle mass losses, due to a decrease of TNF-α receptors in the gastrocnemius muscle. A later study conducted by this group of researchers found that after supplementation with CLA, there was a decrease of the loss of muscle mass, without changing the amount of TNF-α receptor 1 (TNFR1). Thus it appears that this receptor is not actively contributing to the increase of muscle proteolysis in cachexia [6]. Studies by our group showed the effect of CLA supplementation on parameters of lipid metabolism in cachetic rats. Supplementation of CLA was not able to reverse the lipid oxidation in the liver, decreased by cachexia. Moreover, the CLA supplementation promoted greater weight loss and greater accumulation of fat in the liver (hepatic steatosis) and in the plasma [7].

Due to the lack of works explaining the mechanisms which CLA supplementation inhibits muscle proteolysis and the possible role of pro-inflammatory cytokines (such as TNF-α), more researches are still necessary. Despite these found results, CLA appears to be not a good supplement in patients with cachexia.
